# Parental Height Differences Predict the Need for an Emergency Caesarean Section

**DOI:** 10.1371/journal.pone.0020497

**Published:** 2011-06-29

**Authors:** Gert Stulp, Simon Verhulst, Thomas V. Pollet, Daniel Nettle, Abraham P. Buunk

**Affiliations:** 1 Department of Psychology, University of Groningen, Groningen, The Netherlands; 2 Department of Behavioral Biology, University of Groningen, Groningen, The Netherlands; 3 Centre for Behaviour and Evolution, Institute of Neuroscience, Newcastle University, Newcastle, United Kingdom; 4 Royal Netherlands Academy of Arts and Sciences, Amsterdam, The Netherlands; Durham University, United Kingdom

## Abstract

More than 30% of all pregnancies in the UK require some form of assistance at delivery, with one of the more severe forms of assistance being an emergency Caesarean section (ECS). Previously it has been shown that the likelihood of a delivery via ECS is positively associated with the birth weight and size of the newborn and negatively with maternal height. Paternal height affects skeletal growth and mass of the fetus, and thus might also affect pregnancy outcomes. We hypothesized that the effect of newborn birth weight on the risk of ECS would decrease with increasing maternal height. Similarly, we predicted that there would be an increase in ECS risk as a function of paternal height, but that this effect would be relative to maternal height (i.e., parental height differences). We used data from the Millennium Cohort Study: a large-scale survey (N = 18,819 births) with data on babies born and their parents from the United Kingdom surveyed 9 to 12-months after birth. We found that in primiparous women, both maternal height and parental height differences interacted with birth weight and predicted the likelihood of an ECS. When carrying a heavy newborn, the risk of ECS was more than doubled for short women (46.3%) compared to tall women (21.7%), in agreement with earlier findings. For women of average height carrying a heavy newborn while having a relatively short compared to tall partner reduced the risk by 6.7%. In conclusion, the size of the baby, the height of the mother and parental height differences affect the likelihood of an ECS in primiparous women.

## Introduction

Obstructed labor, a failure to progress due to a mismatch between fetal size and the mother's pelvis [1], accounts for 8% of maternal deaths worldwide. Only a minor part of these maternal deaths, i.e. the death of a woman during or shortly after pregnancy [2], occur in the developed world, but obstructed labor is nonetheless a common obstetrical problem. For example, in England more than 30% of all pregnancies require some form of assistance at delivery [3], of which an emergency Caesarean section (ECS) is the most common form (12.7% of all deliveries).

Short maternal stature is associated with adverse pregnancy outcomes, such as stillbirths [4], low birth weight newborns [5], low APGAR scores (a quick assessment of health directly after delivery, based on Appearance, Pulse, Grimace, Activity and Respiration; [6]), and perinatal mortality [7]. Despite having smaller neonates [5], [8], shorter mothers are also at a higher risk for obstructed labor, resulting in an assisted delivery, in particular ECS [2], [9]. Obstructed labor is related to the narrower pelvises of shorter women [10]–[12], through which the head (i.e. cephalopelvic disproportion) or shoulders [13], [14] of the baby is hindered.

Fetus size is also a well-known risk factor for obstructed labor. Heavier and larger newborns increase the likelihood of difficult deliveries (such as an ECS [9], [15]–[20]) or assisted deliveries resulting from shoulder dystocia [14], [20], [21]. A short woman with a heavy and/or large newborn seems particularly at risk for obstructed labor [15], [22]–[24]. In contrast, for taller women, for whom the increased size of the newborn is less likely to lead to obstructed labor [22], [24], a low birth weight newborn seems more predictive of adverse pregnancy outcomes [15]. In the latter situation, operative deliveries are more a result of fetal distress, preeclampsia, or fetal malformations, rather than size-related obstetrical problems [15].

Although the effects of maternal height and birth weight on ECS risk are well established, it is currently unknown whether or not there is an effect of paternal height on the likelihood of having an ECS. Paternal height may influence pregnancy outcomes, as it has a positive effect on neonatal body size [25], [26]. Whereas the height of the mother is especially associated with the size of the newborn through the adiposity of the fetus, the height of the father predicts skeletal growth and fat-free mass of the newborn [25]–[28]. Specifically, research has shown an effect of paternal height on neonatal fat-free mass, but not on fat mass [25], [26], on the length of the baby [26], [28], on neonatal bone mineral content [29], on placental volume [30], and on head circumference [26], [28]. This is relevant because the skeletal structure of the baby is more predictive of birth problems than birth weight [9], [24]. For instance, head circumference is more important in predicting problems at delivery than birth weight [23], [24]. The effect of paternal height on the structural size of the baby may therefore affect the risk for adverse pregnancy outcomes.

Much of the research on size and complications at birth in humans is mirrored by research on obstetric complications in animal research. In cattle, feto-pelvic disproportion, the disproportion between calf size and the size of the birth canal of the cow is the major cause of problems at birth [31]–[33]. In line with the findings on humans, both the size of the cow as well as the size of the calf is a determinant of difficult delivery [31]–[33]. Furthermore, the sire also affects this risk, as pairing cows to sires bred for heavy birth weight calves (versus low birth weight calves; [31], [32]) and sires bred for meat (which are bigger, versus bred for dairy; [34]) increases the risk of difficult delivery. Additionally, as found in humans, the skeletal size of the calf seems more important than the birth weight of the calf for the risk of difficult delivery [32].

In this study, our aim was to test the hypothesis that in addition to maternal height and birth weight, paternal height also affects the risk of ECS. We use the Millennium Cohort Study (MCS) to test this hypothesis. In line with previous findings [15], [22]–[24], we predict that maternal height would interact with birth weight, such that a relatively short woman with a heavy newborn would be most at risk. Furthermore, we extend earlier findings and hypothesize that paternal height also influences the risk for ECS, but that the effect of paternal height would be dependent on the height of the mother. We predict that with increasing parental height differences, the risk for ECS would increase.

## Materials and Methods

The Millennium Cohort Study (MCS) is a survey that gathered information from the parents of 18,819 babies born in the year 2000/2001 in the United Kingdom. Interviews were carried out when the babies were around 9–12 months. Detailed information on the pregnancy and birth was collected as well as anthropometric (maternal and paternal height, age, and birth weight), social and economic information (all self-reported) from the mother and where possible from the father. Self-reported measures of height have been shown to be very reliable in women of reproductive age [35]. The sample was selected from a random sample of electoral wards, disproportionately stratified to ensure adequate representation of all four regions of the UK, areas with higher minority ethnic populations, and deprived areas. The overall response rate was 68% [36]. We used the first Wave of data from the MCS.

For the analyses presented here, we only included White parents for which height data were available who had their first, singleton child (of which the birth weight was available), leaving 4,365 cases. Only White parents were included in the analyses as ethnicity relates to maternal pelvic size, which might influence the risk of ECS [8]. We chose to include only first births, because parity has been shown to be a strong determinant of ECS [18], [37]. This was also evident in our sample, as primiparous women had an average risk of 27%, whereas parous women only had a risk of 9% for an ECS. In addition, obstetrical problems resulting from the large size of the newborn are largely confined to primiparous women [18], [37]. For instance, when delivering a macrosomic baby (i.e. an extremely heavy newborn; >4.5 kg), 39.8% of primiparous women had a normal vaginal delivery, whereas 24.2% had an ECS [37]. In contrast, 81% of multiparous women had a normal vaginal delivery when delivering a macrosomic baby, and only 5.7% had an ECS [37]. Therefore, we restricted our sample to primiparous women.

We performed logistic regressions on our key dependent variable; whether the delivery was normal (i.e. vaginal without complications) or by ECS, leaving in total 3,165 cases. We excluded Caesarean delivery on request (N = 266), assisted breech delivery (N = 9), assisted forceps (N = 376), assisted vacuum extraction (N = 503), water births (N = 11) and other problems without specification (N = 5). However, including these cases (i.e. resulting in a dependent variable vaginal without complications versus any form of assistance) did not change our key results. To examine the effects of maternal and paternal height on birth weight, we performed a linear regression. All analyses were performed in SPSS 17.0.

Occurrence of the various pregnancy outcomes in the Millennium cohort was comparable to national statistics. In our entire sample the occurrence of a normal vaginal delivery and ECS were 68.5% and 12.2% respectively, whereas the national statistics for England for 2000 to 2001 are 66.6% and 12.7% respectively [3].

## Results

### Descriptive statistics

Table 1 provides descriptive statistics of the entire cohort as well as our restricted sample of White couples with singleton, first births for which information on maternal and paternal height and birth weight of the newborn was available (see Table S1 for more descriptive statistics on the sample used for our analyses). As expected, maternal and paternal height were positively correlated, indicating that taller women had taller partners (Pearson r = 0.11; p<0.0001; N = 3,165). Furthermore, taller mothers and fathers had heavier newborns, as both maternal and paternal height positively and independently affected the birth weight of the newborn, with the maternal effect being 66% stronger than the paternal effect (Table 2).

**Table 1:** Characteristics (mean ± standard deviation or %) of the entire cohort and the sample used for our analyses.

	Entire sample		Restricted sample	
		N		N
Maternal height (cm)	163.5±7.0	18,217	164.4±6.9	3,165
Paternal height (cm)	177.8±7.4	12,617	178.7±7.4	3,165
PHD^a^ (cm)	14.1±9.2	12,617	14.3±9.5	3,165
Birth weight (kg)	3.34±0.6	18,484	3.34±0.6	3,165
Delivery outcomes:				
Normal delivery	68.5%	12,666	73.1%	2,314
Emergency CS	12.2%	2,260	26.9%	851
Planned CS	9.4%	1,742		
Other forms of assistance^b^	9.9%	1,828		

The sample used for analyses was White parents (for which height data were available) who had their first, singleton child (of which birth weight was available) through a normal vaginal delivery or an emergency Caesarean section.

a PHD; Parental height differences (paternal minus maternal height).

b Other forms of assistance were: assisted breech delivery, assisted forceps, assisted vacuum extraction, water births and other problems without specification.

**Table 2:** Linear regression parameter estimates of the effects of maternal height and paternal height on birth weight.

	B (± s.e.)	β
Intercept	−1.48*10^−1^ (±3.23*10^−1^)	
Maternal height	1.32*10^−2^ (±1.46*10^−3^)***	0.158
Paternal height	7.41*10^−3^ (±1.37*10^−3^)***	0.095
N	3,165	

Maternal and paternal height in centimeters, birth weight in kilograms.

***p<0.0001.

### Effects of birth weight

**Figure 1:**
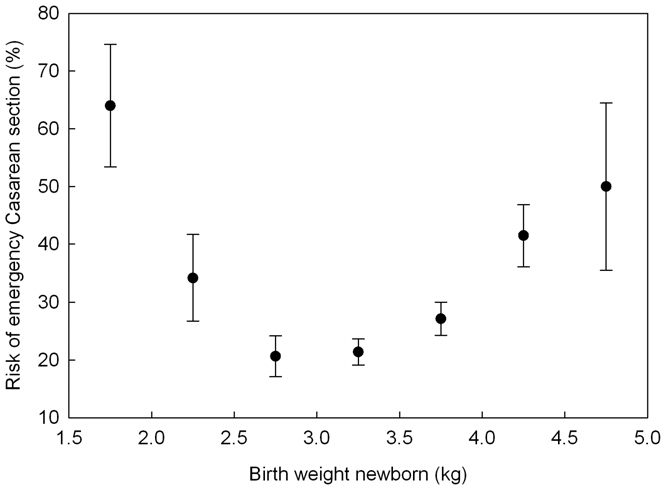
The effect of birth weight (means and 95% confidence intervals of raw data) on the risk of ECS. Birth weight in bins of 0.5 kg and bins lower than 2.5 and higher than 4.5 kg were pooled. The confidence interval was determined using the Agresti-Coull method [43].

Logistic regression revealed a quadratic effect of birth weight on the likelihood of having an ECS: both low and high birth weight newborns had an increased risk for ECS compared to average weight newborns (Table 3A; Figure 1). The lowest risk of 21.8% (the minimum of the quadratic curve) was found at a birth weight of 3.1 kg, which was 0.2 kg below average.

**Table 3:** Logistic regression parameter estimates (± s.e.) of the effects of maternal height, height^2^, birth weight, birth weight^2^, parental height differences and their interactions on the probability of an emergency Caesarean section.

Model	A	B	C	D	E	F
Intercept	4.46 (±6.60*10^−1^)***	54.25 (±15.03)***	46.50 (±15.21)**	44.16 (±15.40)**	114.75 (±29.03)***	116.53 (±29.01)***
Birth weight	−3.75 (±4.14*10^−1^)***	−4.03 (±4.20*10^−1^)***	3.76 (±1.67)*	4.23 (±1.69)*	4.63 (±1.72)**	1.36 (±2.31)
Birth weight^2^ a	6.14*10^−1^ (±6.47*10^−2^)***	6.76*10^−1^ (±6.61*10^−2^)***	7.30*10^−1^ (±6.87*10^−2^)***	7.25*10^−1^ (±7.03*10^−2^)***	7.32*10^−1^ (±7.06*10^−2^)***	7.10*10^−1^ (±7.14*10^−2^)***
Mat. Height		−5.51*10^−1^ (±1.83*10^−1^)**	−6.15*10^−1^ (±1.84*10^−1^)***	−6.03*10^−1^ (±1.86*10^−1^)***	−1.45 (±3.50*10^−1^)***	−1.40 (±3.50*10^−1^)***
Mat. height^2^ a		1.51*10^−3^ (±5.57*10^−4^)**	2.21*10^−3^ (±5.75*10^−4^)**	2.22*10^−3^ (±5.80*10^−4^)**	4.76*10^−3^ (±1.07*10^−3^)***	4.44*10^−3^ (±1.08*10^−3^)***
Mat. height * Birth weight^a^			−4.95*10^−1^ (±1.03*10^−2^)***	−5.22*10^−1^ (±1.05*10^−2^)***	−5.49*10^−1^ (±1.07*10^−2^)***	−3.60*10^−1^ (±1.39*10^−2^)**
PHD				5.21*10^−3^ (±5.70*10^−3^)	−2.21 (±5.61*10^−1^) ***	−2.28 (±5.62*10^−1^) ***
Height*PHD					2.61*10^−2^ (±6.89*10^−3^)***	2.64*10^−2^ (±6.90*10^−3^)***
Height^2^*PHD					−7.65*10^−5^ (±2.14*10^−5^)***	−7.85*10^−5^ (±2.14*10^−5^)***
PHD * Birth weight						2.07*10^−2^ (±9.79*10^−3^)*
N	3,275	3,275	3,275	3,165	3,165	3,165

Height in centimeters, weight in kilograms. PHD is parental height differences ( = paternal height−maternal height).

*p<0.05;

**p<0.01;

***p<0.001 (significance based on Wald test statistic with df = 1).

a We also ran models which included all two-way interactions with maternal height^2^ and birth weight^2^. None of these terms were significant (all p>0.12). We always included the underlying (interaction with the) linear term when including a(n interaction with a) squared term in the model.

### Effects of maternal height

**Figure 2:**
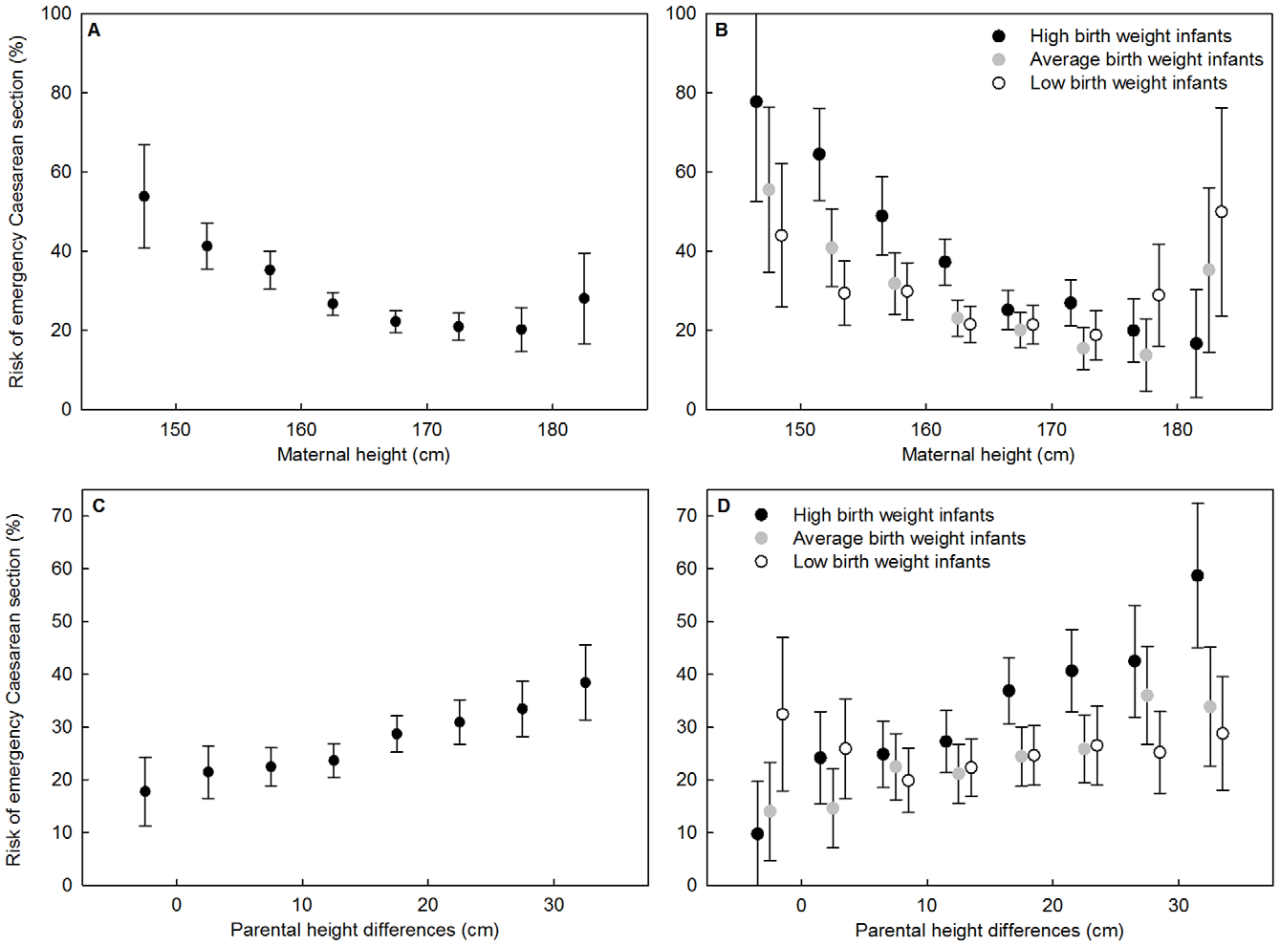
The effect of maternal height, parental height differences and birth weight on ECS risk. The effects (means and 95% confidence interval of raw data) are shown for (a) maternal height, (b) maternal height and birth weight (c) parental height differences and (d) parental height differences and birth weight. Height is divided into bins of 5 cm (bins lower than 145 for maternal height and −5 cm for parental height differences and higher than 180 and 35 cm were pooled) and birth weight was divided into tertiles. The confidence interval was determined using the Agresti-Coull method [43].

Controlling for birth weight, maternal height had a negative effect on the occurrence of ECS. Shorter women were more likely to have had ECS compared to taller women, and this was a decelerating pattern as indicated by a significant quadratic effect of height (Table 3B; Figure 2A). Maternal height interacted with birth weight (Table 3C; Figure 2B), indicating that the risk resulting from the size of the newborn depended on the height of the mother. To illustrate these findings, table 4A provides model predictions for the interaction between maternal height and birth weight. As expected, the highest risk for an ECS arises when short women carry heavy babies. Short women (below mean −1 s.d.) were more than twice as likely to need an ECS (46.3% versus 21.7%) than tall women (above mean +1 s.d.) when carrying a heavy newborn (above mean +1 s.d.). Generally, with increasing birth weight the risk of ECS also increased, but in tall women the risk of having ECS when carrying an average weight newborn was marginally lower compared to when having a light weight newborn (respectively 16.6% and 18.7%; Table 4A, see Table S2 and Figure S1 for model predictions across the entire range of female height).

**Table 4:** Model predictions for the risk (%) of an emergency Caesarean section for A short, average height, and tall mothers and, B average height women with small, average, and large parental height differences for low, average and high birth weight newborns.

		Birth weight newborn				
A		Low	Average	High	RR^a^	OR^a^
Maternal height	Short	24.5	29.4	46.3	1.89	2.66
	Average	19.7	20.5	30.6	1.55	1.80
	Tall	18.7	16.6	21.7	1.16	1.20
	RR^b^	1.31	1.78	2.13		
	OR^b^	1.41	2.10	3.11		

B		Low	Average	High	RR^a^	OR^a^
Parental height differences	Small	19.2	18.6	25.9	1.35	1.47
	Average	18.9	19.7	29.1	1.54	1.76
	Large	18.8	20.9	32.6	1.73	2.09
	RR^c^	0.98	1.12	1.26		
	OR^c^	0.97	1.16	1.38		

Short and small refers to mean − s.d., average refers to mean, and tall and large refers to mean + s.d.. Relative risks (RR) and Odds ratios (OR) are calculated based on the percentages.

a Comparison between high and low birth weight newborns.

b Comparison between short and tall mothers.

c Comparison between large and small parental height differences.

### Effects of parental height differences

Having established that effects of previously identified risk factors (i.e. maternal height and birth weight) on ECS risk are present in the Millennium Cohort Study, we extended the analyses to examine the effects of parental height differences (PHD; paternal minus maternal height) on ECS risk. Logistic regression revealed that, when controlling for maternal height, birth weight and their interactions, there was no main effect of PHD on ECS risk (Table 3D). PHD did, however, affect the risk of ECS, as it significantly interacted with the squared effect of maternal height (Table 3E). With increasing PHD, the risk of an ECS increased (Figure 2C), but the effect of PHD was restricted to women of average height and tall women (Table 3E; see Table S2 and Figure S2 for model predictions of the effect of PHD in short, average height and tall women). There was no effect of PHD in short women, most likely because the risk for ECS in these women was already very high (i.e. a ceiling effect; Figure S2).

In addition to the interaction of PHD with maternal height, PHD also interacted with birth weight (Table 3F, Figure 2D). With increasing PHD the risk of ECS increased, but only when the mother was carrying heavy newborns or newborns of average weight but not when carrying relatively light newborns (Figure 2D). Table 4B provides model predictions for the effect of PHD and birth weight on the occurrence of an ECS for average height women (see Table S2 and Figure S3 for model predictions of the effect of PHD when mothers carry light, average weight and high birth weight newborns). Average height women were most at risk for an ECS (32.6%) when carrying a heavy newborn and having a relatively tall partner (large PHD; Table 4B). The lowest observed risk for average height women was 18.6%, when having small PHD and a baby of average weight.

Having a relatively tall compared to short partner increased the risk for ECS in average height women when carrying a heavy (from 25.9% to 32.6%) or average weighing newborn (from 18.6% to 20.9%; Table 4B). For average height women carrying light weight newborns, increasing PHD hardly changed the likelihood of an ECS (from 19.2% to 18.8%).

The effect of the interactions between maternal height, birth weight and PHD on the risk of ECS remained significant after controlling for maternal and paternal age, self-perceived health, socio-economic status, education, household income, sex of the baby, and gestation time (see Table S3 for parameter estimates). Newborns with low or high birth weight probably increase ECS risk for different reasons, but when excluding low birth weight newborns (below 2.5 kg; [18], [22]) from the analysis the results were very similar (Table S4). This suggests that the effects documented are not driven by newborns with very low birth weights.

### Effects of maternal height and parental height differences independent of birth weight

Given that birth weight is obtained only after birth, and can hence not serve as practical predictor of ECS risk in a clinical setting, we performed additional analyses in which we excluded birth weight (and the interactions with it) to obtain clinical relevant estimates of the effects of maternal height and parental height differences. In line with the results above, logistic regressions revealed a significant squared effect of maternal height (Table 5A). A woman of average height has a 24.9% chance of having ECS at the birth of her first child. Women one standard deviation below average have a risk of 32.7% (an increase of 7.8%), whereas women one standard deviation above average 21.0% (a decrease of 3.9%). There is a 1.56 (32.6%/21.0%) greater probability of ECS for short compared to tall mothers.

**Table 5:** Logistic regression parameter estimates (± s.e.) of the effects of maternal height, height^2^, parental height differences and their interactions on the probability of an emergency Caesarean section.

Model	A	B	C
Intercept	49.56 (±14.54)***	47.53 (±14.70)**	100.85 (±28.07)***
Height	−5.73*10^−1^ (±1.77*10^−1^)**	−5.57*10^−1^ (±1.79*10^−1^)**	−1.19 (±3.34*10^−1^)***
Height^2^	1.61*10^−3^ (±5.38*10^−4^)**	1.58*10^−3^ (±5.43*10^−4^)**	3.48*10^−3^ (±9.91*10^−4^)**
PHD		7.54*10^−3^ (±5.54*10^−3^)	−1.74 (±5.55*10^−1^)**
Height*PHD			2.09*10^−2^ (±6.79*10^−3^)**
Height^2^*PHD			−6.19*10^−5^ (±2.10*10^−5^)**
N	3,275	3,165	3,165

Height in centimeters, weight in kilograms. PHD is parental height differences ( = paternal height−maternal height).

**p<0.01;

***p<0.001 (significance based on Wald test statistic with df = 1).

Similarly, the interaction between maternal height and parental height differences (PHD) remained significant when excluding birth weight from the analyses (Table 5C). A woman of average height with an average PHD, has a 24.7% chance of having undergone ECS. Having a PHD one standard deviation below average would reduce this risk to 22.6% and a PHD difference one standard deviation above average would increase the risk to 26.9% for a woman of average height (see Table S5 for model predictions of the effect of PHD for short, average height and tall women). There is a 1.19 (26.9%/22.6%) higher probability of ECS for women of average height with larger compared to smaller partner height differences. Thus, women with a relatively tall partner were more likely to have had an ECS, also when effects of newborn weight are ignored.

## Discussion

In this study, we have shown that the size of the newborn, the height of the mother and parental height differences all predict the risk of an emergency Caesarean section in primiparous women. We replicated the finding that both lower and higher birth weight newborns increase the risk of ECS [15], [17], [18]. Whereas the increased risk for heavy weight newborns is likely to be a consequence of size-related obstetrical problems, the increased risk for low birth weight newborns may be more a result of fetal distress, preeclampsia and fetal malformations rather than size-related obstetrical problems [15]. In line with previous studies [9], [38], we also found that shorter women are at a higher risk for an ECS and that with increasing height the decrease in risk became progressively weaker. Maternal height interacted with birth weight: shorter women were especially susceptible to the effect of newborn weight on ECS risk (in line with earlier studies [15], [22]–[24]). When carrying a heavy newborn (one SD above average weight), short women were more than twice as likely to need an ECS than tall women. For taller women, for which the overall risk of ECS is lowest, the increased size of the baby had little effect on ECS risk and a low birth weight newborn seems more predictive of an adverse pregnancy outcome for reasons discussed above.

Furthermore, to our best knowledge, we documented for the first time that the height of the father, specifically parental height differences, also affected the occurrence of ECS. The effect of the parental height differences on ECS was, however, dependent on the height of the mother and the birth weight of the newborn. Women with tall compared to short partners relative to their own height, had an increased ECS risk when carrying an average weight and heavy newborn, but not when carrying a light weight newborn, and this effect was most pronounced in average height and tall women. For shorter women, the overall ECS risk was highest, and parental height differences had little additional influence on ECS risk. Average height and tall women giving birth to a heavy newborn were at higher risk when their partners were relatively tall (respectively 32.6% and 25.0%) compared to short (respectively 25.9% and 19.4%). As the structural size of the baby has been shown to be more important in predicting problems at birth than birth weight [24] and the height of the father predicts the structural size rather than the adiposity of the fetus [25], [26], having a tall partner relative to the height of the mother, will result in a relatively larger (in structural size) fetus for that mother, which in turn increases the risk for ECS. Particularly, having a high birth weight newborn with large PHD suggests that the structural size of this baby is large, which causes most problems for the delivery. The mismatch between the size of the fetus and the mother results in adverse pregnancy outcomes [15], [22]–[24]. Unfortunately, in our sample no data were available on the structural size (e.g. head circumference, length) of the newborn, and we thus have no finer grained measures to further substantiate our results.

The finding that differences in height between father and mother influence pregnancy outcomes partly explains the increased risk of assisted deliveries for shorter women. Shorter women have partners who are on average much taller than themselves and with increasing female height, the difference in height between partners decreases strongly. Thus, the higher risk for adverse pregnancy outcomes for shorter women is partly due to the fact that they are more likely to have a partner much taller than themselves.

The finding that parental height differences predict the need for ECS is also consistent with a study investigating cross-national variation in height differences between the sexes [39]. This study found that “maternal death caused by deliveries and complications of pregnancy (a variable known to be size related) could be a key determinant explaining variation in sexual stature dimorphism [sex differences in height] across populations” ([39]; p 2529). According to these authors, tall mothers would more likely survive childbirth, which would result in females getting taller relative to males, thereby decreasing the average height differences between the sexes. Based on our data, the reverse association is also likely: the cross-national variation in height differences between the sexes might explain the variation in maternal deaths caused by deliveries and complications during pregnancy. When average height differences between the sexes are large, fetuses would be relatively large for the mothers carrying them, resulting in more complications at birth.

A potential limitation of our study is the nature of the sample, in particular the oversampling of individuals from deprived areas. However, controlling for socio-economic status with several indicators (household income, National Vocational Qualifications (NVQ levels) / National Statistics Socio-economic Classifications (NS-SEC)) did not change our results, which suggests that the effects of maternal and parental height differences on the risk of ECS are independent of socioeconomic status. Another limitation is that the data are self-reported, through interviews approximately 9 months after birth. However, national health statistics regarding rates of assisted deliveries for England in 2000–2001 are comparable to the rates in our sample. In addition, it seems unlikely that there is a systematic error in reporting problems at birth associated with height: there is little reason to assume that women of a certain height or women with a partner of a certain height would be more likely to over- or underreport complications such as an ECS.

The incidence of ECS may be an imperfect index of obstructed labor, as a physician bias related to maternal height might have occurred [40]. The need for assistance at delivery may be overrated for short women, due to physicians' expectations of difficulty at delivery. This potential bias might have influenced our results for the risk of ECS for short women, but it seems an unlikely explanation for the effects of the parental height differences on ECS risk as this effect is also present for women of average height and for tall women.

From a functional perspective, documented preferences for partner height among men and women (e.g. [41], [42]) are consistent with our finding that parental height differences predict the likelihood of ECS. Whereas women prefer men taller, but not too tall, men prefer women shorter but not too short. Our results suggest that these mate preferences could be adaptive as a male partner too tall or a female partner too short will both result in an increased risk for obstructed labor.

## Supporting Information

Figure S1 Model predictions for the effect of maternal height on the risk (%) of an emergency Caesarean section for mothers carrying low (mean − s.d.), average (mean) and high (mean + s.d.) birth weight newborns.
(TIF)

Figure S2 Model predictions for the effect of parental height differences on the risk (%) of an emergency Caesarean section for short (mean− s.d.), average (mean) and tall (mean + s.d.) mothers carrying an average birth weight newborn.
(TIF)

Figure S3 Model predictions for the effect of parental height differences on the risk (%) of an emergency Caesarean section for average height mothers carrying low (mean − s.d.), average (mean) and high (mean + s.d.) birth weight newborns.
(TIF)

Table S1 Characteristics (mean ± standard deviation or %) of the sample used for our analyses.
(DOC)

Table S2 Model predictions for the risk (%) of an emergency Caesarean section for low (mean − s.d.), average (mean) and high (mean + s.d.) birth weight newborns having (a) short (mean − s.d.), average height (mean), and tall (mean + s.d.) mothers and (b) small (mean − s.d.), average (mean), and large (mean + s.d.) parental height differences for short, average height (c) and tall mothers (d).
(DOC)

Table S3 Logistic regression parameter estimates (± s.e.) of the effects of maternal height, height^2^, parental height differences (PHD), birth weight, their interactions, and control variables on the probability of an emergency Caesarean section.
(DOC)

Table S4 Logistic regression parameter estimates (± s.e.) of the effects of maternal height (cm), height^2^, birth weight (kg), birth weight^2^, parental height differences (cm) and their interactions, on the probability of an emergency Caesarean section when light birth weight newborns (<2.5 kg) are excluded.
(DOC)

Table S5 Model predictions for the risk (%) of an emergency Caesarean section for short, average height, and tall mothers with small, average and large parental height differences (PHD).
(DOC)
