# Supplementary material for: Parental Height Differences Predict the Need for an Emergency Caesarean Section
Source: PLoS One. 2011 Jun 29;6(6):e20497. doi: 10.1371/journal.pone.0020497 (PMC3126796; doi:10.1371/journal.pone.0020497)
Supplement: Table S1 — Characteristics (mean ± standard deviation or %) of the sample used for our analyses. (DOC) [file pone.0020497.s004.doc]

Table S1.

|  | Mean ± sd/% | Min | Max | N |
| --- | --- | --- | --- | --- |
| Maternal height (cm) | 164.4 ± 6.9 | 121.9 | 203.2 | 3,165 |
| Paternal height (cm) | 178.7 ± 7.4 | 132.1 | 213.36 | 3,165 |
| PHD^a^ (cm) | 14.3 ± 9.5 | -35.6 | 68.6 | 3,165 |
| Birth weight (kg) | 3.34 ± 0.6 | 0.71 | 6.78 | 3,165 |
| Delivery outcomes |  |  |  | 3,165 |
| Normal delivery | 73.1% |  |  | 2,314 |
| Emergency CS | 26.9% |  |  | 851 |
| Age mother (yrs) | 27.1 ± 5.6 | 13 | 48 | 3,165 |
| Age father (yrs) | 30.1 ± 6.3 | 15 | 57 | 3,160 |
| Household income |  |  |  | 3,008 |
| 0-3,100 £ | 1.0% |  |  | 30 |
| 3,100-10,400 £ | 11.4% |  |  | 344 |
| 10,400-20,800 £ | 34.6% |  |  | 1042 |
| 20,800-31,200 £ | 26.5% |  |  | 797 |
| 31,200-52,000 £ | 19.1% |  |  | 575 |
| > 52,000 £ | 7.3% |  |  | 220 |
| Health mother^b^ |  |  |  | 3,165 |
| Excellent | 36.7% |  |  | 1,161 |
| Good | 52.0% |  |  | 1,645 |
| Fair | 9.6% |  |  | 305 |
| Poor | 1.7% |  |  | 54 |
| Health father^b^ |  |  |  | 3,165 |
| Excellent | 36.1% |  |  | 1,141 |
| Good | 51.6% |  |  | 1,633 |
| Fair | 10.6% |  |  | 334 |
| Poor | 1.8% |  |  | 57 |
| NS-SEC mother^c^ |  |  |  | 3,069 |
| Managerial and professional occupations | 39.3% |  |  | 1,205 |
| Intermediate occupations | 22.5% |  |  | 689 |
| Small employers and own account workers | 3.2% |  |  | 99 |
| Lower supervisory and technical occupations | 5.3% |  |  | 162 |
| Semi-routine and routine occupations /  Never worked and long-term unemployed | 29.8% |  |  | 914 |
| NS-SEC father^c^ |  |  |  | 3,115 |
| Managerial and professional occupations | 41.8% |  |  | 1,302 |
| Intermediate occupations | 5.8% |  |  | 180 |
| Small employers and own account workers | 10.3% |  |  | 322 |
| Lower supervisory and technical occupations | 16.4% |  |  | 512 |
| Semi-routine and routine occupations /  Never worked and long-term unemployed | 25.7% |  |  | 799 |
| Education mother^d^ |  |  |  | 3,128 |
| None of these qualifications | 5.6% |  |  | 174 |
| NVQ Level 1 | 7.0% |  |  | 219 |
| NVQ Level 2 | 30.1% |  |  | 942 |
| NVQ Level 3 | 17.9% |  |  | 560 |
| NVQ Level 4 | 35.1% |  |  | 1,097 |
| NVQ Level 5 | 4.3% |  |  | 136 |
| Education father^d^ |  |  |  | 3,115 |
| None of these qualifications | 9.3% |  |  | 234 |
| NVQ Level 1 | 7.5% |  |  | 955 |
| NVQ Level 2 | 30.7% |  |  | 553 |
| NVQ Level 3 | 17.8% |  |  | 935 |
| NVQ Level 4 | 30.0% |  |  | 147 |
| NVQ Level 5 | 4.7% |  |  |  |
| Gestation time (days) | 278.1 ± 14.7 | 170 | 296 | 3,165 |
| Sex baby |  |  |  | 3,165 |
| Male | 52.2% |  |  | 1,651 |
| Female | 47.8% |  |  | 1,514 |

The sample used for analyses was White parents (for which height data were available) who had their first, singleton child (of which birth weight was available) through a normal vaginal delivery or an ECS

^a^ PHD; Parental Height Differences (paternal minus maternal height)

^b^ Self-perceived health.

^c^ The National Statistics Socio-economic Classification. The reference category was ‘managerial and professional occupations’ (<http://www.ons.gov.uk/about-statistics/classifications/current/ns-sec/index.html>)

^d^ National Vocational Qualifications. (<http://www.direct.gov.uk/en/EducationAndLearning/QualificationsExplained/DG_10039029>)
