# Supplementary material for: Parental Height Differences Predict the Need for an Emergency Caesarean Section
Source: PLoS One. 2011 Jun 29;6(6):e20497. doi: 10.1371/journal.pone.0020497 (PMC3126796; doi:10.1371/journal.pone.0020497)
Supplement: Table S2 — Model predictions for the risk (%) of an emergency Caesarean section for low (mean − s.d.), average (mean) and high (mean + s.d.) birth weight newborns having (a) short (mean − s.d.), average height (mean), and tall (mean + s.d.) mothers and (b) small (mean − s.d.), average (mean), and large (mean + s.d.) parental height differences for short, average height (c) and tall mothers (d). (DOC) [file pone.0020497.s005.doc]

Table S2.

|  |  | Birth weight newborn | | |  |  |
| --- | --- | --- | --- | --- | --- | --- |
| A |  | Low | Average | High | RR^a^ | OR^a^ |
| Maternal height | Short | 24.5 | 29.4 | 46.3 | 1.89 | 2.65 |
|  | Average | 19.7 | 20.5 | 30.6 | 1.55 | 1.79 |
|  | Tall | 18.7 | 16.6 | 21.7 | 1.16 | 1.21 |
|  | RR^b^ | 1.31 | 1.78 | 2.13 |  |  |
|  | OR^b^ | 1.41 | 2.10 | 3.11 |  |  |
| B Short women | | |  |  |  |  |
| Parental height differences | Small | 27.4 | 29.5 | 42.6 | 1.55 | 1.96 |
|  | Average | 26.0 | 29.8 | 45.1 | 1.73 | 2.34 |
|  | Large | 24.7 | 30.1 | 47.7 | 1.93 | 2.78 |
|  | RR^c^ | 0.90 | 1.02 | 1.12 |  |  |
|  | OR^c^ | 0.87 | 1.03 | 1.23 |  |  |
| C Average height women | | |  |  |  |  |
| Parental height differences | Small | 19.2 | 18.6 | 25.9 | 1.35 | 1.47 |
|  | Average | 18.9 | 19.7 | 29.1 | 1.53 | 1.75 |
|  | Large | 18.8 | 20.9 | 32.6 | 1.73 | 2.09 |
|  | RR^c^ | 0.98 | 1.12 | 1.25 |  |  |
|  | OR^c^ | 0.97 | 1.16 | 1.38 |  |  |
| D Tall women | | |  |  |  |  |
| Parental height differences | Small | 17.9 | 15.3 | 19.4 | 1.09 | 1.11 |
|  | Average | 17.7 | 16.3 | 22.1 | 1.25 | 1.32 |
|  | Large | 17.5 | 17.3 | 25.0 | 1.43 | 1.57 |
|  | RR^c^ | 0.98 | 1.13 | 1.29 |  |  |
|  | OR^c^ | 0.97 | 1.16 | 1.38 |  |  |

Relative risks (RR) and Odds ratios (OR) are calculated based on the percentages

^a^ Comparison between high and low birth weight newborns

^b^ Comparison between short and tall mothers

^c^ Comparison between high and low parental height differences
