# Supplementary material for: Parental Height Differences Predict the Need for an Emergency Caesarean Section
Source: PLoS One. 2011 Jun 29;6(6):e20497. doi: 10.1371/journal.pone.0020497 (PMC3126796; doi:10.1371/journal.pone.0020497)
Supplement: Table S3 — Logistic regression parameter estimates (± s.e.) of the effects of maternal height, height2, parental height differences (PHD), birth weight, their interactions, and control variables on the probability of an emergency Caesarean section. (DOC) [file pone.0020497.s006.doc]

Table S3.

| Intercept | 55.13 ± 16.75** | 121.60 ± 33.14*** |
| --- | --- | --- |
| Birth weight (kg) | 3.77 (± 1.83)* | 3.95*10^-1^ (± 2.68 *10^-1^) |
| Birth weight^2^ | 7.80*10^-1^ (± 8.34*10^-2^)*** | 7.80*10^-1^ (± 8.34*10^-2^)*** |
| Height (cm) | -7.55*10^-1^ (± 2.01*10^-1^)*** | -1.48 (± 3.99*10^-1^)*** |
| Height^2^ | 2.65*10^-3^ (± 6.25*10^-4^)*** | 4.63*10^-3^ (± 1.22*10^-4^)*** |
| Height * Birth Weight | -5.16*10^-2^ (± 1.12*10^-2^)*** | -3.24*10^-2^ (± 1.61*10^-2^)* |
| PHD (cm) |  | -2.51 (± 5.90 *10^-1^)*** |
| Height * PHD |  | 2.93*10^-2^ (± 7.21 *10^-3^)*** |
| Height^2^ * PHD |  | -8.83*10^-5^ (± 2.24 *10^-5^)*** |
| PHD * Birth weight |  | 4.88*10^-2^ (± 8.86 *10^-3^)*** |
| Age mother (yrs) | 1.02 *10^-1^ (± 1.01*10^-2^)*** | 1.08 *10^-1^ (± 1.29*10^-2^)*** |
| Age father (yrs) |  | 1.49*10^-3^ (± 1.04 *10^-2^) |
| Household income^b^ |  |  |
| 3,100-10,400 £ | -5.32*10^-1^ (± 5.08*10^-1^) | -4.72*10^-1^ (± 5.60*10^-1^) |
| 10,400-20,800 £ | -4.70*10^-1^ (± 4.86*10^-1^) | -4.46*10^-1^ (± 5.37*10^-1^) |
| 20,800-31,200 £ | -4.49*10^-1^ (± 4.90*10^-1^) | -4.75*10^-1^ (± 5.41*10^-1^) |
| 31,200-52,000 £ | -5.05*10^-1^ (± 4.95*10^-1^) | -4.85*10^-1^ (± 5.46*10^-1^) |
| > 52,000 £ | -3.07*10^-1^ (± 4.10*10^-1^) | -2.79*10^-1^ (± 5.62*10^-1^) |
| Health mother^c^ |  |  |
| Good | 1.79*10^-1^ (± 9.71*10^-2^) | 1.77*10^-1^ (± 1.02*10^-1^) |
| Fair | 3.37*10^-1^ (± 1.61*10^-1^)* | 3.77*10^-1^ (± 1.67*10^-1^)* |
| Poor | 5.06*10^-1^ (± 3.43*10^-1^) | 4.51*10^-1^ (± 3.59*10^-1^) |
| Health father^c^ |  |  |
| Good |  | 9.66*10^-3^ (± 1.01*10^-1^) |
| Fair |  | -1.19*10^-2^ (± 1.70*10^-1^) |
| Poor |  | -8.80*10^-1^ (± 4.78*10^-1^) |
| NS-SEC mother^d^ |  |  |
| Intermediate occupations | 1.42*10^-1^ (± 1.26*10^-1^) | 1.96*10^-1^ (± 1.31*10^-1^) |
| Small employers and own account workers | -1.03*10^-1^ (± 2.63*10^-1^) | -3.10*10^-3^ (± 2.74*10^-1^) |
| Lower supervisory and technical occupations | 3.06*10^-1^ (± 2.23*10^-1^) | 1.12*10^-1^ (± 2.30*10^-1^) |
| Semi-routine and routine occupations /  Never worked and long-term unemployed | 6.52*10^-2^ (± 1.43*10^-1^) | 1.20*10^-1^ (± 1.49*10^-1^) |
| NS-SEC father^d^ |  |  |
| Intermediate occupations |  | -1.59*10^-1^ (± 2.13*10^-1^) |
| Small employers and own account workers |  | -3.32*10^-2^ (± 1.79*10^-1^) |
| Lower supervisory and technical occupations |  | -3.86*10^-2^ (± 1.49*10^-1^) |
| Semi-routine and routine occupations /  Never worked and long-term unemployed |  | 3.45*10^-2^ (± 1.48*10^-1^) |
| Education mother^e^ |  |  |
| NVQ Level 1 | -6.76*10^-2^ (± 2.93*10^-1^) | -2.53*10^-1^ (± 3.14*10^-1^) |
| NVQ Level 2 | 3.08*10^-1^ (± 2.47*10^-1^) | -3.10*10^-2^ (± 2.63*10^-1^) |
| NVQ Level 3 | 7.78*10^-3^ (± 2.58*10^-1^) | -9.18*10^-3^ (± 2.75*10^-1^) |
| NVQ Level 4 | -6.26*10^-3^ (± 2.56*10^-1^) | -3.59*10^-2^ (± 2.74*10^-1^) |
| NVQ Level 5 | -6.38*10^-2^ (± 3.20*10^-1^) | -4.59*10^-2^ (± 3.48*10^-1^) |
| Education father^e^ |  |  |
| NVQ Level 1 |  | 3.64*10^-3^ (± 2.63*10^-1^) |
| NVQ Level 2 |  | 2.97*10^-1^ (± 2.01*10^-1^) |
| NVQ Level 3 |  | 3.62*10^-1^ (± 2.17*10^-1^) |
| NVQ Level 4 |  | 7.46*10^-2^ (± 2.21*10^-1^) |
| NVQ Level 5 |  | 1.43*10^-1^ (± 2.98*10^-1^) |
| Gestation time (days) | 1.68*10^-3^ (± 4.51*10^-3^) | 1.74*10^-3^ (± 4.69*10^-3^) |
| Sex baby^f^ | -1.32*10^-1^ (± 8.97*10^-2^) | -1.50*10^-1^ (± 9.32*10^-2^) |
| N | 2,972 | 2,817 |

*p<0.05, **p<0.01, ***p<0.001 (significance based on Wald test statistic with df=1).

^a^ This interaction was not significant (p>0.72)

^b^ The reference category was income bin 0-3,100 £

^c^ Self-perceived health. The reference category was ‘Excellent’

^d^ The National Statistics Socio-economic Classification. The reference category was ‘managerial and professional occupations’ (<http://www.ons.gov.uk/about-statistics/classifications/current/ns-sec/index.html>)

^e^ National Vocational Qualifications. The reference category was ‘none of these qualifications’ (<http://www.direct.gov.uk/en/EducationAndLearning/QualificationsExplained/DG_10039029>)

^f^ The reference category was male
