# Supplementary material for: Parental Height Differences Predict the Need for an Emergency Caesarean Section
Source: PLoS One. 2011 Jun 29;6(6):e20497. doi: 10.1371/journal.pone.0020497 (PMC3126796; doi:10.1371/journal.pone.0020497)
Supplement: Table S4 — Logistic regression parameter estimates (± s.e.) of the effects of maternal height (cm), height2, birth weight (kg), birth weight2, parental height differences (cm) and their interactions, on the probability of an emergency Caesarean section when light birth weight newborns (<2.5 kg) are excluded. (DOC) [file pone.0020497.s007.doc]

Table S4.

| Intercept | 46.51 (± 17.22)** | 123.12 (± 30.34)*** |
| --- | --- | --- |
| Birth weight | 7.93 (± 2.34)*** | 4.67 (±3.16) |
| Birth weight^2^ | 4.84*10^-1^ (± 1.65*10^-1^)** | 4.34*10^-1^ (± 1.71*10^-2^)* |
| Mat. Height | -7.08*10^-1^ (± 2.04*10^-1^)*** | -1.56 (± 3.66*10^-1^)*** |
| Mat. height^2^ | 2.64*10^-3^ (± 6.36*10^-4^)*** | 5.00*10^-3^ (± 1.14*10^-3^)*** |
| Mat. height * Birth weight | -6.33*10^-2^ (± 1.45*10^-2^)*** | -4.43*10^-1^ (± 1.93*10^-2^)* |
| RPH |  | -2.63 (±5.82*10^-1^) *** |
| Height*RPH |  | 3.05*10^-2^ (± 7.12*10^-3^)*** |
| Height^2^*RPH |  | -9.14*10^-5^ (± 2.21*10^-5^)*** |
| RPH * Birth weight |  | 3.03*10^-2^ (±1.38*10^-2^)* |
| N | 3,048 | 2,944 |

PHD is parental height differences (=paternal height – maternal height)

*p<0.05; **p<0.01; ***p<0.001 (significance based on Wald test statistic with df=1).
