# Supplementary material for: Parental Height Differences Predict the Need for an Emergency Caesarean Section
Source: PLoS One. 2011 Jun 29;6(6):e20497. doi: 10.1371/journal.pone.0020497 (PMC3126796; doi:10.1371/journal.pone.0020497)
Supplement: Table S5 — Model predictions for the risk (%) of an emergency Caesarean section for short, average height, and tall mothers with small, average and large parental height differences (PHD). (DOC) [file pone.0020497.s008.doc]

Table S5.

|  |  | Parental height differences | | |  |  |
| --- | --- | --- | --- | --- | --- | --- |
|  |  | Small | Average | Large | RR^a^ | OR^a^ |
| Maternal height | Short | 32.1 | 33.2 | 34.4 | 1.07 | 1.11 |
|  | Average | 22.6 | 24.7 | 26.9 | 1.19 | 1.26 |
|  | Tall | 19.7 | 21.6 | 23.8 | 1.21 | 1.27 |
|  | RR^b^ | 1.63 | 1.54 | 1.45 |  |  |
|  | OR^b^ | 1.93 | 1.80 | 1.68 |  |  |

Short and small refers to mean – s.d., average refers to mean, and tall and large refers to mean + s.d.. Relative risks (RR) and Odds ratios (OR) are calculated based on the percentages

^a^ Comparison between large and small partner height differences

^b^ Comparison between short and tall mothers
